# The Use of Medicinal Plants by Migrant People: Adaptation, Maintenance, and Replacement

**DOI:** 10.1155/2012/807452

**Published:** 2011-11-10

**Authors:** Patrícia Muniz de Medeiros, Gustavo Taboada Soldati, Nélson Leal Alencar, Ina Vandebroek, Andrea Pieroni, Natalia Hanazaki, Ulysses Paulino de Albuquerque

**Affiliations:** ^1^Departamento de Biologia, Área de Botânica, Universidade Federal Rural de Pernambuco, R. Dom Manoel de Medeiros, S/N, Dois Irmãos, 52171-900 Recife, PE, Brazil; ^2^Instituto de Ciências Ambientais e Desenvolvimento Sustentável, Universidade Federal da Bahia, Campus Edgard Santos, Estrada do Barrocão, Morada Nobre, 47800-000 Barreiras, BA, Brazil; ^3^Universidade Federal do Piauí, Campus Amílcar Ferreira Sobral, BR 343, Km 3,5, Meladão, 64800-000 Floriano, PI, Brazil; ^4^The Institute of Economic Botany, The New York Botanical Garden, 2900 Southern Boulevard, Bronx, NY 10458, USA; ^5^University of Gastronomic Sciences, Via Amedeo di Savoia 8, Pollenzo, 12060 Bra, Italy; ^6^Departamento de Ecologia e Zoologia (ECZ), Centro de Ciências Biológicas (CCB), Universidade Federal de Santa Catarina, Campus Universitário, Cidade Universitária, 88040-900 Florianopolis, SC, Brazil

## Abstract

Given the importance of studying the knowledge, beliefs, and practices of migrant communities to understand the dynamics of plant resource use, we reviewed the scientific literature concerning the use of medicinal plants by migrant populations engaged in international or long-distance migrations. We considered the importance of two processes: (1) adaptation to the new flora of the host country (i.e., substitution and incorporation of plants in the pharmacopoeia) and (2) continued use and acquisition of the original flora from migrants' home countries (i.e., importation, cultivation, and/or continued use of plants that grow in both host and home environments). We suggest that, depending on the specific context and conditions of migration, different processes that determine the use and/or selection of plants as herbal medicines may become predominant.

## 1. Introduction

It is estimated that in 2010 approximately 214 million people lived in a location other than their country of origin [1], which corresponds to approximately 3% of the human population. This does not take into account internal migrations within a country, which are also important processes. The study of migrant people's health-seeking behavior can aid in understanding (1) the effects of cultural contacts in the dynamics of medicinal plant knowledge (2) the different ways in which therapeutic resources are perceived and used by the people, and (3) the biological and cultural contexts that determine the behavior of social groups [2–8].

There are numerous reasons why people leave their homeland. However, one of the central arguments in migration studies is that, upon reaching a new location, the newcomers face difficulties they did not previously experience (such as the lack of knowledge about the sociocultural and/or natural environment). Similarly, new opportunities absent in the homeland can also be experienced, such as an improved availability of certain health resources or land for agriculture [2, 9]. As stated by Lacuna-Richman [9] *“neither difficulties arise without relief, nor limitations without opportunities*.*”*

One of the issues that arise from the study of plant use behavior in migrant groups is how this use change after migration and which changes occur in their therapeutic practices [10]. In this study, we consider the arguments of Volpato et al. [5], who proposed that two forces drive the traditional medical knowledge of migrant people: (1) the adaptation of their ethnomedical system to the new environment, in which the plant resources formerly used (in the home country) are being replaced with ones from the new environment and (2) the development of strategies to use and obtain the original plant medicines, either through cultivation or collection of species that occur in both environments, importation and marketing, or maintaining contact with relatives and friends from the place of origin. Regardless of the type of strategy followed by migrant groups, the present study assumes that cultures are not static [11]. Therefore, it is understood that they have the ability to define and actively influence the environment in which they exist.

Pieroni and Vandebroek [10] collected several chapters describing the ethnobiology of migrant groups and highlighted that there is little data available on this subject so far. These authors emphasized the migration of groups of people from the tropics to temperate countries, or intratropical and intratemperate migrations. In this paper, we continue to expand on this overview by examining the role of the processes triggered by migration that involve the use of medicinal plants in light of the available literature. We seek to categorize medicinal plant use strategies by migrant groups and discuss, with examples from the available literature, in which situations each of these strategies stands out. The referred strategies are divided into two groups: (1) adaptation of the ethnomedical system to the new flora of the host country and (2) acquisition of the original plants from the home country. Therefore, this paper was carried out to show evidence from the literature concerning our main hypothesis that the choice of the two type of strategy for a given migrant population will highly depend on factors such as (1) the degree of floristic and environmental similarity between home and host country, (2) variation in the prevalence of health conditions between home and host country, (3) degree of contact with local populations in the host country, (4) the involvement of social networks and the degree of contact between migrants and their homeland, (5) the ability and ease to acquire plants via importation (presence of commercial routes and plant entrance laws in the host country). Each of these factors will be further discussed in the present paper. It is also important to emphasize that our main focus is on international or long-distance migrations and we are not considering processes and patterns of internal migrations.

## 2. Adapting the Ethnomedical System to the New Flora of the Host Country

### 2.1. Replacement or Substitution of Species from the Home Environment with New Species from the Host Environment

When faced with a new environment that contains new plant species, migrants can, consciously or subconsciously, develop strategies to preserve the structure of their medical system by replacing plants from their place of origin with plants from the new environment [12]. This replacement process has been described in several studies of migrant populations [3, 8, 12–19]. In addition, we highlight some studies that specifically deal with African-Brazilian communities (i.e., people of African descent who went to Brazil during the period of slavery) [20–27]. 

When they arrived in Brazil, African slaves were forced to conform to the new environment. However, it seems that the biogeographic similarities of the continent of origin and the new environment made it easier for Africans to succeed in replacing plants with Brazilian species because both areas have dry forests and wetlands with similar physiognomies [22]. Some studies reinforced this hypothesis, observing that the African descendants maintained a local plant classification system very similar to the one from their places of origin [22, 25]. In many cases, the replacement of African plants with Brazilian was accompanied by transposing common names from the former to the latter. Such is the case with *niamba*, which refers to species of the genus* Cannabis* in Africa; in Brazil, the name was designated to the species *Vitex agnus-castus* L. Such substitution is supported by the morphological similarities between the leaves of these two taxa. Thus, Brazilian and African species may possess the same vernacular name, because these names were given to the Brazilian plants due to their similarities with African species [25, 26]. These observations led to the following question by Camargo [21]: what are the criteria that lead to the replacement of a plant from a place of origin with one from a new environment? Among the factors that can play a role in the process of replacement are taxonomic, morphological, chemical, and sensory characteristics of plants, as well as the influence of contact with local people [9].

Given the taxonomic similarity of the Brazilian and African floras, African species of the genera *Bauhinia, Kalanchoe, Vernonia*, and* Peperomia* were easily replaced by Brazilian plants of the same genera [22]. Species with phylogenetic proximity may contain similar compounds, guaranteeing their usefulness for the same purpose. However, given the importance of symbolism and ritual in African-Brazilian ethnomedicine, many species are likely to be replaced on the basis of their morphological similarity rather than because of their shared physiological or chemical effects. In the case of other replaced species, merely a sensory similarity, rather than a morphological correspondence, may be in order. Voeks [22], in comparing Yoruba practices with those of the Brazilian Candomblé, stated that because the bitter taste of plants plays a role in treating spiritual disorders, taxonomically distant bitter plants can be replaced by and even inherit the same names as the African originals. Thus, bitterness may be an inherent feature that facilitates and directs the replacement of plants in the host environment. In specialized herbal shops in the United States, called *botánicas*, Latino immigrants can purchase a herbal remedy called *salvia* or sage (*Pluchea carolinensis *(Jacq.) G. Don), which has a smell that reminds of European sage (*Salvia officinalis* L.) [28], even though there are distinctive features to the smell of both species. *P. carolinensis *is a shrub that grows naturally throughout the West Indies and from Mexico to northern South America and southern Florida. Interestingly, these species belong to very different plant families: *P. carolinensis *is an Asteraceae, whereas *S. officinalis* belongs to the Lamiaceae. However, their similarity in smell is probably close enough for these species to have acquired the same common name [28]. 

Finally, social and cultural pressures may require the replacement of species used as medicinal resources. Volpato et al. [6] investigated the preparation of a typical medical-religious beverage by Haitians living in Cuba and found that this drink is primarily prepared with *Artemisia absinthium* L, along with other macerated herbs, as an alcoholic drink. The authors state that some changes are occurring in the preparation of the drink and that the prejudice of the Cubans towards Haitian immigrants was the driving force behind these changes. Haitians were seen as less respected by Cuban society and as followers of Voodoo, a cultural practice that was frowned upon in the country. As a result, today that drink is used more often in culinary applications or as an aperitif rather than for magic and religious purposes, resulting in a change of species composition. Specifically, the trend has been to incorporate additional herbs and to reduce the amount of *A. absinthium* to make the drink less bitter.

It appears that plant replacement patterns in pharmacopoeia are strongly influenced by the characteristics of the host environment. Migrant populations can encounter three different scenarios: (a) the population migrates to a region far from the homeland that is virtually uninhabited [12, 19], (b) the population migrates to a region that is occupied by a traditional and usually native community [9], or (c) the migrant population moves to multicultural urban centers (e.g., Ceuterick et al. [3]). The involvement of each of these three scenarios may result in different strategies and degrees of substitution of plants.

When migrant populations settle in new places, they become intensively dependent on the new ecosystem. If there are no means to acquire plants from their original ecosystem, the population tends to replace unavailable medicinal plants with plants found in the surrounding flora. Inta et al. [12] compared the medicinal flora of two Akha populations (ethnic groups scattered in Southeast Asia) of which one migrated to China and the other to Thailand. The authors noted that the environment plays an important role in medicinal plant selection since the majority of the medicinal plants were not shared between the two communities; this is because most of the medicinal flora of the migrant population belongs to the settled ecosystem rather than the region of origin. Although all ecosystems are somehow different from one another, it is reasonable to hypothesize that significant differences between the host and home ecosystems can lead to important differences in plant use when it is difficult to acquire plants from the home country. 

When migrants come in touch with local or indigenous communities, it is expected that the substitution of plants will be influenced to some degree by the prevalent practices of these peoples. Lacuna-Richman [9], in studying the use of nontimber forest products by Visayan people who migrated to Palawan (both in the Philippines), observed that most of the forest species of the new environment were initially unknown to the migrant people. Furthermore, they found that such species were presented to them by the native people or former migrants, usually in joint events of resin collection. Other studies have described the influence of indigenous peoples on African descendents in Brazil [23, 26].

### 2.2. Incorporation of New Plant Species or New Uses for Known Species in the Ethnopharmacopoeia

We consider the use of a plant species an incorporation when a new plant species is inserted in the pharmacopeia of migrant people without replacing a plant that was already known and used in the group's place of origin, thus occupying a niche in the pharmacopeia that was not previously considered. Although it is not easy to acknowledge whether a plant is being substituted or incorporated, in certain situations, a group selects a medicinal resource to treat a previously unknown therapeutic demand. This factor distinguishes replacement from incorporation for the purposes of our study. It can be either that a new plant species is incorporated into the pharmacopoeia, or that a new medicinal use for a formerly used species (in the home country) is incorporated in the new environment. For example, this is the case for plants used to treat high levels of cholesterol among immigrants from the Dominican Republic in New York City, as investigated by Vandebroek et al. [15]. In that study, the authors found that the use of plants for high cholesterol, such as *Avena sativa *L. (oats)*, Ananas comosus *(L.) Merr. (pineapple)*, Cucumis sativus *L. (cucumber), and* Apium graveolens *L. (celery), was not corroborated by the literature data on the use of these medicinal plants in the Dominican Republic. However, even though no literature data could be found that corroborated theses uses, the plant species belong to the contemporary pharmacopoeia in the Dominican Republic, and several of these same medicinal uses were confirmed during a comparative ethnobotanical study in the Dominican Republic in 2006, a year after recording the data in New York City. However, not all uses were corroborated in both host and home environments, and some of these uses probably represent true incorporations, such as the use of cucumber and celery to treat hypertension in New York City (unpublished data). In Brazil, the ritual use of *jurema* (*Mimosa tenuiflora* (Willd.) Poir) was probably incorporated into African traditions through contact with and influence of the indigenous Brazilians [27].

In the case of medicinal plants, an important factor that directs the incorporation of species into the medical systems of migrant people without replacement is the occurrence of new diseases that did not exist in the home environment. When faced with new diseases, migrant people can either develop strategies to treat those conditions with plants from their place of origin or from their new environment. Food plants that were incorporated by migrant groups can be taken as a reference. In a study of migrants from India in the United States, Palaniswamy [16] classified the diets of respondents as traditional, semitraditional, semiwesternized, or westernized, according to the consumption frequency of some items commonly consumed in the country of origin. The study found that dietary modification in the United States omitted foods with antihyperglycemic properties. This creates a concern regarding the incidence of diabetes, which is proportionally higher among US Indians in comparison with Caucasians.

New eating habits that are different from traditional ones can thus contribute to an increased incidence of diseases such as diabetes or even nutritional deficiencies, conditions for which migrant groups may not have ethnobotanical pharmacopoeias. Because migrant populations tend to abandon traditional diets that could prevent such conditions, the incorporation of a set of specific therapeutic practices for disease treatment is required. Such practices can be based on either conventional (biomedical) systems or the incorporation of certain plants from the new location that have already been identified as effective to treat these conditions by the host culture. In this case, trial and error and contact with local people may play important roles.

One central question in medical ethnobiology is how people diagnose recently appearing diseases, especially those that are difficult to identify, such as hypertension and diabetes. One possibility is that migrant people exchange information about those diseases with people from the host environment, which are used to deal with these diseases. But this needs to be further investigated. Outside the context of migration, some studies have described the use of herbal treatments for recently appearing diseases in local populations [29], but few studies have focused on how people treat previously unknown diseases with regard to the use of plants. For migrant populations, this theme is particularly relevant because they come into contact with a new environment that can host a range of new diseases hitherto unknown to them. In addition, it is important to consider that migrants often do not have easy access to health services, due to their immigrant status, lack or deficiency of such services, or other barriers, including those of language and culture [30, 31].

### 2.3. Abandonment of Plants or Practices from the Original Pharmacopoeia

Unlike replacement or incorporation, some plants or practices associated with them may simply be abandoned when a group migrates to a new environment. Abandonment of practices occurs when, for example, groups that originally inhabited areas with certain diseases migrate to areas where these diseases do not occur. van Andel and van't Klooster [18] reported that half the population of Surinam migrated to The Netherlands between 1972 and 1996; in this case, it became clear that the migratory population was no longer susceptible to malaria or other tropical infections. Ceuterick et al. [3] observed that plants previously used for the treatment of parasitic diseases in Colombia were no longer used for that purpose in London because the conditions that lead to people contracting such diseases were negligible in the inner city relative to the locations from which the Colombians migrated. 

 One reason for plant abandonment can be the impossibility of obtaining plants from the home country. This scenario can be due to the lack of social or commercial networks between the former and current countries, or even due to strict importation laws in the host country. Ceuterick et al. [3], for example, noticed that some Colombian migrants in London refrain from importing medicinal plants since it is common to associate Colombians with drugs and also because of the strict British importation laws. In New York City, some medicinal plants originating from the Caribbean, such as the Dominican endemic *Melocactus lemairei* (Monv. ex Lem.) Miq. ex Lem. are subject to CITES protection because of their ecological vulnerable status and are prohibited from importation.

When these difficulties to acquire certain plants lead to substitution, it can be argued that there is a loss in plant use but the practice previously associated with that species still remains. But, when hard-to-obtain plants are not substituted by other plants easily found in the host country, then not only the previously used plants are lost, but also the practices associated with them (e.g., plant use for treating disease X). As a consequence, these practices may become forgotten or even substituted by the use of allopathic alternatives.

## 3. Acquisition of Original Plants (from the Home Countries)

### 3.1. The Cultivation and Use of Wild Plants That Occur in Both the Host and the Home Environment

One method to bring plants closer from the original source is to cultivate these plants from the home country in the new environment or to use plants that spontaneously grow in both environments and that were already part of the group's pharmacopoeia prior to migration [5, 6, 22, 32]. For example, Indian migrants in Connecticut (USA) listed several culturally important plants they were growing in their home gardens, including mango, banana, curry leaf, holy basil, mint, jasmine, hibiscus, eucalyptus, oleander, gardenia, pomegranate, marigold, and *Bougainvillea*, despite difficulties of growing these plants in a temperate climate, bringing them from India, or obtaining them from specialty stores [16]. Given these difficulties, this strategy of acquiring plants is less often pursued as compared to other strategies, such as substitution and importation. According to Voeks [22], factors such as the coevolution of plant species with specific pollinators, coupled with frequent dioecy in many tropical species, reduce the possibility of acclimatization in new environments. In the case of African-Brazilian communities, seeds of African species such as *Garcinia kola* were brought to Brazil in several attempts at cultivation that failed due to the lack of fructification in the New World [22].

The *Tifey* example studied by Volpato et al. [6] also shows how the cultivation of plants in a new location was an important method for maintaining traditional practices. The authors found that some of the Haitian migrants they interviewed cultivated in their yards the primary species used in the production of *Tifey*. Therefore, despite some prejudice related to the use of this drink by the host culture, Haitian elders preserve its use by cultivating the main species that it consists of (*Artemisia absinthium*).

On the other hand, some species grow spontaneously in both the migrant people's place of origin and the new environment. Thus, there are no major obstacles in continuing the use of the same species. For example, Volpato et al. [5] have indicated that the high floristic similarity between Haiti and Cuba enabled Haitian immigrants to collect many of the medicinal plants from their homeland in Cuba. However, an in-depth analysis of the importance of native plants of the host country versus those that grow naturally in both the migrants group's country of origin and host country is often hampered by the lack of information in the literature regarding the geographic distribution of these species. When there exists a greater distance between the source environment and new environment, species with high dispersal potential (e.g., cosmopolitan species) can excel in the pharmacopoeias of migrant peoples. In fact, there is evidence that plants that are considered invasive or weeds are important both in terms of traditional pharmacopoeias and medical effectiveness [33, 34]. It has been noted in many studies that some of the more important medicinal plants for local communities are cosmopolitan plants that are characterized by a high probability of occurring in both the homeland and the new location. There exists evidence in the literature that more accessible or more ubiquitous species tend to be more useful to people [35–37]. 

The importance of invasive species can be quite high in the pharmacopoeias of migrant groups, particularly because the collection of these species may be the easiest way to acquire plants from the place of origin without the need to cultivate, purchase, or travel to the former environment. Such importance of this kind of plants can be observed in the literature from studies around the world. Voeks [22], for example, noted that 63% of the plants used by people of African descent in a region of Bahia (NE Brazil) are considered weeds, and many of these weeds are from the New World. Anthony [24] also noted that certain cosmopolitan or pantropical species, such as *Peperomia pellucida* (L.) Kunth and *Bidens pilosa *L., are used both in Africa and Brazil by people of African origin.

### 3.2. Importation of Plants from the Place of Origin

Certain plants of the migrant group's place of origin may not be readily available fresh in the new environment, due to the inability of natural occupation by these species, the inability of acclimation to the new location or simply because the new location is a major urban center, which reduces the possibility of cultivation or collection. However, when social ties are maintained between the migratory group and their home country, there is a continuous flow of people between the two locations, creating a possibility to acquire plants with relative ease. According to Balick et al. [38], the process of globalization that ensures the migration of people between countries and regions also allows for the importation or purchase of plant resources from their homeland. Thus, globalization creates transnational groups and also enables the exchange of genetic material through either purchase or exchange [38]. For example, van Andel and van't Klooster [18] have stated that roughly 2,000 kg of plant material enters The Netherlands from Suriname every week. However, this exchange of genetic material may face barriers due to the sanitary restrictions between different countries, as reported by Ceuterick et al. [3] in a study of plant use by Latino immigrants in London.

The role of markets, shops, apothecaries, or other places selling traditional medicines including dried or fresh plants, potions, tinctures, or religious objects, as distributors of species originating from source environments has been described in the literature [4, 14, 15, 18, 32, 39, 40]. Studying the role of these stores, Balick et al. [38] concluded that they are extremely important for Latin American immigrants living in New York, USA; these stores make hundreds of different plant species available to migrants, some of which are unique to the customers' countries of origin, enabling migrant groups to maintain their traditional medical systems. 

In the case of migrant communities that move to large multicultural urban centers, the literature indicates that many retain their use of traditional medicine in centers that either contain markets selling traditional medicinal plants [3] or enable access to medicinal plants from their place of origin [6]. Such scenarios have been described in the cases of Surinamese migrants in Amsterdam [18] and migrants from the Dominican Republic [14] and Latin American countries [40] in New York City. In these cases, substitution may play a secondary role since people can easily get plants from their home country. Furthermore, ethnomedical systems based on the use of traditional herbal remedies can largely be replaced or complemented by biomedicine and allopathic remedies [17]. Similar phenomena occur in situations where groups who once lived in less-urbanized areas start to get better access to modern health services, regardless of whether or not they are migrant groups [41–43]. However, the coexistence of medicinal plant-based health seeking strategies with healing practices based on allopathic remedies is nowadays very paradigmatic of many health systems all over the world, not only in urban areas [44].

Another possibility to acquire these plants arises when people return to visit family and friends in their home countries, thus transporting local plants from the home country to their host country [4, 32]. Usually, the plants that are transported represent dried or processed material to prevent problems with agricultural inspection when entering the host country again. 

Volpato et al. [19] studied the adaptation of traditional Sahrawi (nomadic tribes and shepherds traditionally from Western Sahara) in refugee camps in Algeria, located in desert environments and with scarcity of plant resources. Less than 2% of 57 plant species could be obtained locally, due to the extremely arid conditions. Thus, the Sahrawi depend on several social networks to obtain traditional remedies from their homeland. In this case, the continued use of traditional medicine represents an important element for maintaining the cultural identity of the group by maintaining a link with their places of origin [19].

In studying the use of medicinal plants by Colombians who had migrated to London, Ceuterick et al. [3] found that remedies that contained fresh produce as an essential ingredient were generally abandoned and replaced with fresh material that was available in London.

Depending on the type of contact that migrant people have with their place of origin, importation can be quite an important strategy. In New York City, 100 of the 112 most frequently mentioned medicinal plants (89%) by Dominican immigrants could be readily obtained either in *botánicas* (herbal stores that import plants transnationally) or in supermarkets (unpublished data). Turkish migrants in Cologne (Germany), for example, acquire half of their ingredients directly from Turkey through collection or purchase from markets in their country of origin [32]. Another significant portion of the materials is purchased in Cologne, through Turkish markets importing plants from Turkey [32]. Pieroni et al. [4] also observed that most medicinal plants used by Pakistani migrants in Bradford were sold in Pakistani markets in Northern England, while some species were also acquired during visits to relatives in Pakistan. The importance of markets can also be observed in the work performed by Waldstein [39]. She studied a group of Mexican immigrants living in Athens, USA, and concluded that none of the plants known and used in their ethnomedical systems were directly brought by the migrants from their country of origin, but rather were purchased from small Mexican grocery stores that, in turn, imported the materials from Mexico. Ceuterick et al. [8] also found that 34% of plant species used by Peruvian and Bolivian migrants in London were acquired in supermarkets, while another 16% were bought in Latino shops in London.

Another issue that may determine the importance of importation as a strategy for continued plant use is related to legal aspects of plant entrance in a given country. The degree of strictness in the regulation of importation can strengthen or weaken this plant use strategy. Surinamese migrants in Amsterdam, for example, rely heavily on importation because of the flexible entrance laws in The Netherlands, since no permit is needed for importing plants for personal use (except if it is a threatened species), and, although commercial importation requires a permit, all plants sold at a Surinamese medicinal plant market (which was the focus of the study performed by van Andel and van't Klooster [18]) were imported from Suriname.

On the other hand, strictness in plant importation in the United Kingdom makes it difficult for Latino communities to acquire some of their original plants [3, 8]. Peruvian migrants in London, for example, have difficulties in continuing the use of coca (*Erythroxylum coca* Lam.) products because of legal restrictions concerning the importation of this plant species. Although Colombian migrants can acquire their products in supermarkets and Latino shops, their own import from Colombia is less probable because of the British importation laws [3]. Most Northern hemisphere countries including the United States and Europe have strict laws concerning plant importation [45].

Another factor worthwhile mentioning is that it is not always possible to acquire each and any plant species used in the home country. Some species can be endemic or scarce, and international plant traders often consider only the most commonly used and valued plants. Therefore, pharmacopoeias based on endemic and noncommercial or less popular species may have difficulties to count on importation.

## 4. Replacement or Maintenance?

Next, we will try to make a comparison between strategies to acquire medicinal plants based on adaptation to the plants of the new environment and those based on obtaining the original plants. However, it is important to take into account that this was usually not the direct focus of the studies on the pharmacopoeias of migrant people and that we are indirectly analyzing this information based on nonuniform data obtained from the literature.

Some scholars [12, 22] have indicated that many plants used by migrant people are native to their new environments. A study that investigated African descendants in Brazil showed that approximately 49% of the plants used were from the New World, 35% from the Old World and 16% of uncertain origin [22], indicating that New World native plants have been incorporated or replaced in the ethnomedical systems of people of African origin.

A study of Dominican immigrants in New York City related their ethnobotanical data with information available in the literature on medicinal plants used in the Dominican Republic [13]. The authors found that only 29% of the plants used by the migrants were reported in Dominican literature on medicinal plants and attributed this to replacement of a portion of the medicinal plants used. On the other hand, Vandebroek et al. [15], also working with Dominican migrants in New York, found that major medicinal uses in New York, such as flu and common cold, were supported by the Dominican literature, while plants used for minor and “newly acquired or diagnosed” purposes, such as elevated levels of cholesterol, were not (yet) registered in the Dominican literature.

Additional evidence that supports the replacement hypothesis was provided by Inta et al. [12] in their investigation on the use of medicinal plants among the Akha people (from China) living in China and Thailand. Their study showed that only 16 of the 95 recorded plant species (17%) were used both in China and Thailand. This highlights the role of the environment versus the importance of cultural heritage in terms of the forces governing the continued use of traditional species.

However, replacement and incorporation are not always predominant factors in the dynamics of medicinal plant use by migrant people, particularly when migration is directed to a transnational major urban center. Some studies point out that strategies of acquisition of the original material from the home countries are more important factors in medicinal plant use by migrants [3, 32].

Regarding the use of medicinal plants by the Turkish community in Cologne (Germany), Pieroni et al. [32] noted that from the list of species mentioned two-thirds were plants that were used only in the Turkish pharmacopoeia, whereas the remaining third were used in both Turkish and German pharmacopoeias. Similarly, in their study of people of Colombian origin in London, Ceuterick et al. [3] observed that 43% of medicinal plants were reported only in the ethnobotanical literature of Colombia, while 31% were reported in both Colombian and Western literature; thus, 74% of the species were present in the pharmacopoeia of the country of origin. The other species were found exclusively in Western herbal medicine (5%) or were not reported in any literature source (21%).

Pieroni and Gray [46] noted that the vast majority of medicinal plants that are used by Russlanddeutschen (ethnic descendants of Germans who migrated to Russia and returned to Germany) either occurred in both Russia and Germany or only in Russia. Given the complex nature of this migration, accentuated by the departure and return to Germany, this result may indicate (1) that Russlanddeutschen, when they migrated to Russia more than two centuries ago, replaced their remedies by incorporating Russian plants into their pharmacopeia or/and (2) that, after having migrated back to Germany in the last decade, they developed strategies to continue using plants from their Russian original pharmacopoeia without the need for replacement. Thus, two opposite strategies may have occurred. 

Other studies [24, 27] only state that there was replacement of plants with those in the new environment, without mentioning or illustrating the importance of this phenomenon in comparison to other strategies for the selection and use of species, such as importation or cultivation.

Adaptation to the new flora is not always the most important strategy, though it seems to play an important role in the pharmacopoeias of migrant populations. Given the characteristics of the studies cited, we assume that the degree of urbanization and the contact of migrant people with people in the location of origin may influence the types of strategies adopted; specifically, the more urbanized the host location and the higher the degree of contact, the greater the tendency to purchase the original flora, due to either a lack of substitute species (in the case of urban centers) or the ease of obtaining the original plants (in case of continued contact with the people of origin).

## 5. Contact between Western and Traditional Medical Systems: Migration to Urban Centers

An important aspect to consider when analyzing the dynamics of medicinal plant use by migrants in urban centers is the intensity of contact of such groups with biomedicine. For analytical purposes, here we are generally referring to the dominant medical systems in urban centers, as well as the effects of their proximity. In its elemental form, these two medical systems, traditional medicine and biomedicine, present quite different ideologies, concepts, values, and belief systems. Traditional medicine typically considers the whole person and the person's cultural beliefs and values in the healing process. In contrast, biomedicine, synonym here of Western medicine, often operates with a Cartesian approach of separated body systems [47]. There are also contrasts in the way people understand and explain what sickness and health are, which are determined by cultural ideas and belief systems [2]. This contrast determines the choice of healing practices [41], and it can influence how medicinal plants are used by migrants, either distancing them or binding them more tightly to their traditional medical practices.

The coexistence of traditional and Western medical systems can occur, even though this can be conflicting at times. Several examples have been cited in the literature of such coexistence [2, 17, 41, 48] even if some traditional practices are sometimes lost. The stability of coexistence between these medical systems depends strongly on the access to elements of the flora of the migrant culture (e.g., access to markets, cultivation, or direct importation) and socioeconomic factors. According to Nesheim et al. [49], an improvement in the economic status of migrant populations undermines this coexistence because it reduces the dependency of the population on plant resources. On the other hand, retaining some traditional therapeutic practices from their places of origin may be a deliberate choice of migrant groups as a way of reaffirming their cultural identity [10].

If migrant populations do not have access to the plant species that are important to their traditional medical systems, then these systems may become at risk of extinction. Volpato et al. [6] found that Haitian people who migrated to Cuba had difficulties acquiring plants needed to produce *Tifey*. In some cases, other ingredients present in the Cuban flora had to be used as substitutions, which hampered the long-term continuity of the practice.

In general, difficulties in accessing biomedicine, because of its high cost, lack of transportation or available time, or the illegal status of some migrants, encourage the use of traditional medicinal resources. Waldstein [39] evaluated the traditional pharmacopoeia of Mexican immigrants in the USA and noted that, because of the strong influence of Western medicine, there was a coexistence between traditional and Western medical systems. In some cases, a combination of plants and drugs were used to treat disease. Belliard and Ramírez-Johnson [2] observed the same phenomenon when they analyzed the decision-making processes of Mexican women in California. Zapata and Shippee-Rice [7] studied a similar phenomenon in Latin American immigrants living in New England, USA. These authors cited the unawareness about the traditional medicine of these migrants, by medical professionals, difficulty in communication (different languages), and cultural prejudice as obstacles preventing access to biomedicine.

Zenk et al. [50] devised a theoretical model to discuss the events of choice in situations of cultural shock, called the “push and pull" model. According to these authors, external forces that are political, cultural, or economic either encourage or discourage the use of traditional medical systems (including medicinal plants) of specific cultural groups. The forces discussed in the "push and pull" model can be recognized as either positive or negative. As an example, the presence of biomedicine or difficulty in obtaining medicinal plants counteracts the use of traditional medical systems, whereas the absence of biomedicine because of geographical distance or socioeconomic barriers, as well as kinship, values and beliefs, is a driving force toward the use of traditional resources [51].

In some situations, contact with biomedicine results in a hybrid system in which traditional medicine and biomedicine are fused, or systems in which the two approaches are fairly intimately related. The Latin American immigrants in the study of Zapata and Shippee-Rice [7] traditionally consulted with experts of their own medical system, called traditional healers (*curandeiros*). However, since it was nearly impossible to consult with these healers in the New England town to which these people had migrated, immigrants sought cures in biomedicine, by consulting with doctors who most resembled traditional healers (i.e., physicians that were recognized as “naturalists" who prescribed traditional medicine drugs).

## 6. Problems and Challenges in Research with Migrants

One of the factors that should be considered in future studies focusing on migrants and that still lacks understanding is the dynamic nature of resource utilization when migrant groups are no longer subject to a specific disease. For example, a group might migrate to a new location where there are no vectors for a given disease that is endemic in their homeland (e.g., malaria). In this case, it is likely that the plants that were previously used by the group would be dismissed from their pharmacopeias in the new environment. Thus, absence of use of a resource does not reflect, in and of itself, an erosion of knowledge. Rather, it may simply indicate that the species is not longer being used because there is no longer is a demand for its use. 

Some researchers believe that different cultural groups, in addition to developing their own cultural systems, are biologically adapted to their environments. Thus, they may develop an entire metabolic and physiological system that reflects local pressures. That is, different cultures exhibit unique sets of specific biological processes that depend on the environments in which they live and that determine which resources should be either used or avoided. This reasoning has not yet been thoroughly tested or studied though it may allow for a better understanding of the biological determinants of cultural practices.

Regarding the approach used to determine the influence of migration on ethnomedical systems, it is often difficult to figure out how much knowledge about medicinal plants has been lost by migrants in comparison with people from their place of origin because often no baseline data exist about these medical systems prior to migration. Some studies have developed strategies to perform comparisons, including (a) comparing the pharmacopeias of migrant people with the available literature of the place of origin [3, 5, 13, 15, 32, 46] and (b) comparing the pharmacopoeias of migrant people with the pharmacopoeias of the new environment's native people (e.g., Pieroni and Quave [52]). A third strategy would be to simultaneously compare the pharmacopoeias of a migrant group in the new host country with that in its place of origin. 

Some limitations to these approaches are evident, as described below.

- Even in migrant groups' home countries there will be uses reported in the literature that are no longer active nowadays and are therefore not a part of the repertoire of species of the migrants [3, 13]. This means that there was no loss in cultural knowledge *after* migration.
- The fact that a species is used by migrants but is not present in the literature of the place of origin does not mean that this plant was included in the pharmacopeia after migration. It may only represent a gap in the ethnobotanical literature of the place of origin, which did not record representative or contemporary data on the local use of medicinal plants.

These reasons underscore the importance of identifying the origin and distribution of medicinal plants used by migrant populations because such information is an important indicator of the influences of local medicinal plants in these populations. For example, when a migrant group's pharmacopoeia is mostly composed of plants that are native to the host country rather than the home environment, it is likely that there was a process of change and people adapted to the flora of the host country. However, even if this proves to be the case, a comparison with the contemporary pharmacopoeia in the migrant group's home environment is still needed to analyze the proportion of global and cosmopolitan plant species versus native medicinal plants since globalization processes may have already had a profound impact on these pharmacopoeias prior to the process of migration. 

## 7. Conclusions

Depending on the context and conditions of migration, different processes may play a role in the dynamics of medicinal plant use by migrant communities. 

One of the main questions that emerges from this paper is under which circumstances does there exist a higher incidence of replacement and addition of plants to the original ethnopharmacopoeia and which factors are responsible for the opposite effect (acquisition of plants from the home country's pharmacopoeia)?

Several different hypotheses could answer this question. These include the following.

- In environments with similar floristic compositions (i.e., floristic similarity), replacement does not need to occur because migrant people have access to virtually the same diversity of plants as in the place of origin.
- When migration occurs but there is an easy link to the place of origin (i.e., two-way flow), the importation and cultivation of the plants from the place of origin are expected to be more important than practices of substituting medicinal plants.
- When migration is directed at large urban multicultural centers, the following phenomena can occur: (1) if it is easy to obtain plants from the local place of origin through either purchase or cultivation the use of these plants may be favored over replacing/substituting plants; (2) if access to these plants is difficult (e.g., due to availability, accessibility, or economic cost), it is expected that migrants would gradually lose their practice of using medicinal plants; (3) as migrants become more familiar with and better integrated in the host environment's biomedical system, they may prefer this system over their own ethnomedical practices. Research that compares preferences for both systems in first generation migrant groups versus the next generations may shed light on this assumption.
- Replacement will be more important when (1) the species of local origin are morphologically or taxonomically similar to the flora of the place of migration, enabling the use of substitutes with similar characteristics or (2) in the new environment there are communities that make use of (other) medicinal plants and they share this knowledge with the migrant groups.

In summary, we consider that the dynamics of medicinal plant use in migrant populations are intimately influenced by environmental, physical, economic, and sociocultural aspects and that further research is needed to elucidate which factors play a more important role in different situations.
